# The Genetics of Pituitary Adenomas

**DOI:** 10.3390/jcm9010030

**Published:** 2019-12-21

**Authors:** Christina Tatsi, Constantine A. Stratakis

**Affiliations:** Section on Endocrinology and Genetics, *Eunice Kennedy Shriver* National Institute of Child Health and Human Development (NICHD), National Institutes of Health, Bethesda, MD 20892, USA; christina.tatsi3@nih.gov

**Keywords:** gene, pituitary, tumor

## Abstract

The genetic landscape of pituitary adenomas (PAs) is diverse and many of the identified cases remain of unclear pathogenetic mechanism. Germline genetic defects account for a small percentage of all patients and may present in the context of relevant family history. Defects in *AIP* (mutated in Familial Isolated Pituitary Adenoma syndrome or FIPA), *MEN1* (coding for *menin*, mutated in Multiple Endocrine Neoplasia type 1 or MEN 1), *PRKAR1A* (mutated in Carney complex), *GPR101* (involved in X-Linked Acrogigantism or X-LAG), and *SDHx* (mutated in the so called “3 P association” of PAs with pheochromocytomas and paragangliomas or 3PAs) account for the most common familial syndromes associated with PAs. Tumor genetic defects in *USP8*, *GNAS*, *USP48* and *BRAF* are some of the commonly encountered tissue-specific changes and may explain a larger percentage of the developed tumors. Somatic (at the tumor level) genomic changes, copy number variations (CNVs), epigenetic modifications, and differential expression of miRNAs, add to the variable genetic background of PAs.

## 1. Introduction

Pituitary adenomas (PAs) are common lesions in the adult population presenting in 15–20% of cadavers or radiologic findings and constitute approximately 10% of all intracranial tumors [1,2]. PAs are rarer in the pediatric population identified in 0.2% of children undergoing brain imaging [3]. Although most of the identified PAs are incidental findings without the need for intervention, some may present as clinically significant because they secrete hormones or cause symptoms from compression or invasion of surrounding tissues [4].

The etiology of PAs is diverse, and more than half of them do not have an identified genetic cause. In certain cases, however, germline or somatic genetic defects are associated with the formation of PAs. Additional genetic changes found in PAs, such as copy number variations (CNVs), methylation changes and miRNA abnormalities have also been investigated as being potentially involved in the pathogenesis, presentation and behavior of these tumors, especially with regard to aggressiveness and response to treatment.

This review focuses on the current knowledge of the genetic findings in PAs at the germline (Table 1 and Table 2) and somatic (Table 3) level. Additional information on the genomic profile of PAs can derive from expression studies, methylation analyses and miRNA changes, which are briefly mentioned in the current review.

**Figure 1:**
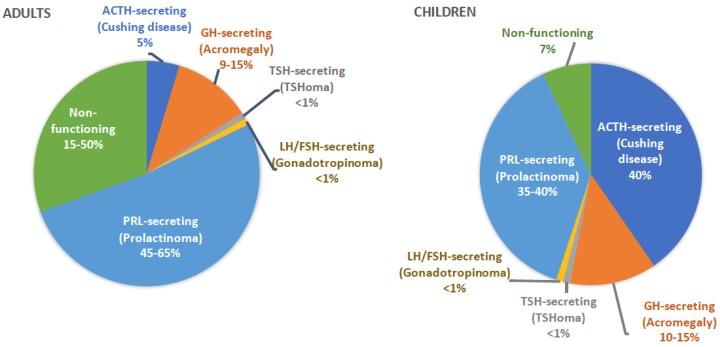
Types and frequency of PAs based on their functional status in adult and pediatric patients.

PAs can be classified based on their functional status (Figure 1). In adults, most PAs are prolactin (PRL)-secreting, while non-functioning adenomas represent the second most common type. In children, depending on the study population, ACTH- and PRL-secreting adenomas are the most commonly encountered subtypes [5]. PAs may also be classified as sporadic (95% of cases), when relevant family history is not present, or familial (5% of cases), when additional family members with similar or relevant disorders are identified [6].

## 2. Germline Defect and Associated Syndromes

### 2.1. Familial Isolated Pituitary Adenomas (FIPA)

The term Familial Isolated Pituitary Adenoma (FIPA) is used to describe families with at least two members with pituitary adenomas, with or without other abnormalities [7]. FIPA accounts for 2–4% of all patients with PAs [7]. Tumors within a family can be homologous (of the same subtype) or heterologous (of different subtypes) [7].

Aryl hydrocarbon receptor interacting protein (*AIP*) gene mutations were described as a cause of FIPA in 2006, and they are currently thought to account for almost 15% of all FIPA cases; this number is even higher (up to 75%) when families with GH-secreting adenomas are selected [8,9]. *AIP* gene defects are less common in sporadic pituitary adenomas identified in up to 8% of all cases [10,11]. However, when younger patients (<30 years old) with macroadenomas, or children and adolescents <18 years old are selected, up to 20.5% of cases with sporadic PAs may harbor an *AIP* mutation [12,13,14,15]. The exact mechanism of the pituitary tumorigenesis with *AIP* mutations is not clear. Some evidence suggests that there is interaction of *AIP* in the synthesis of cAMP, and decreased *AIP* activity leads to aberrant cAMP levels, which then affect several pathways involving cell proliferation [16].

Patients with *AIP* mutations commonly present with GH-secreting adenomas; the second most common presentation is that of GH/PRL co-secreting adenomas. It has been often described that PAs with *AIP* mutations have a more aggressive presentation compared to *AIP*-negative tumors: patients often present in younger age, with larger tumors, higher chance of invasion of surrounding tissues, higher risk for apoplexy, and less chance for control with one intervention, especially in the pediatric population [17,18,19,20,21,22].

### 2.2. Multiple Endocrine Neoplasia Syndromes

Multiple endocrine neoplasia (MEN) syndromes are autosomal dominant disorders that present with combination of tumors in at least two endocrine systems [23].

MEN 1 (OMIM#131100) is caused by mutations in the tumor suppressor gene *MEN1* [24] Patients with MEN1 present with anterior pituitary adenomas in approximately 40% of all cases [25,26]. Although pituitary adenomas commonly present in the third to fourth decade of life, they have been described in children as young as 5 years of age [26,27,28,29,30]. Notably, PAs may be the first manifestation of MEN1 syndrome, and evaluation for other related comorbidities may be indicated [25,26,31]. Most of the PAs in MEN1 are PRL-secreting (42–62%) or non-functioning tumors (15–42%), but GH- (6.5–9%) and ACTH-secreting (3–4%) adenomas have also been described in Table 1 [25,26].

Interestingly, PAs in MEN1 have higher chance of co-secreting multiple hormones compared to *MEN1*-negative patients, in up to 39% of cases [27]. PAs in MEN1 are considered more aggressive and at higher risk for resistance to treatment, especially in children with large prolactinomas [32]. Somatic *MEN1* gene mutations are not commonly found in sporadic pituitary tumors [33,34].

MEN2A (OMIM#171400) and MEN2B (OMIM#162300) syndromes, caused by *RET* gene mutations, have rarely been associated with PAs [35]. *RET* gene codes for a transmembrane receptor with tyrosine kinase activity that acts as proto-oncogene [36]. To date, only few case reports have described patients with MEN2A/2B and PAs: GH-secreting (*n* = 1), ACTH-secreting (*n* = 1) and non-functioning (*n* = 1) adenomas [37,38,39]. Furthermore, *RET* gene mutations do not commonly present in patients with isolated sporadic or familial PAs, suggesting that it is a rare cause of pituitary adenomas [40,41,42].

MEN4 (OMIM#610755) is caused by *CDKN1B* gene mutations, which codes for a cyclin-dependent kinase (p27) that regulates cell cycle and progression from G1 to S phase of mitosis [43]. MEN4 is a rare genetic syndrome, accounting for approximately 1.5–3% of patients clinically classified as MEN1, without genetic defects in *MEN1* gene [44,45]. Of the reported index cases to date, five patients have been diagnosed with PAs (two with GH-secreting, two with ACTH-secreting, and one with non-functioning adenoma) [44,45,46,47,48,49]. *CDKN1B* gene mutations in FIPA have been a rare entity and are described in approximately 2% of *AIP*-negative FIPA kindreds [50]. To date, only one patient with isolated sporadic GH-secreting PA has been described in the literature [51].

### 2.3. Carney Complex

Carney complex (CNC) describes the constellation of myxomas, spotty skin pigmentation, and endocrine overactivity [52,53]. Germline mutations of the *PRKAR1A* gene are responsible for more than 70% of cases of CNC (OMIM#160980), whereas few patients may harbor defects at another locus on chromosome 2p16 [54,55]. *PRKAR1A* codes for the type 1 alpha regulatory subunit of the protein kinase A (PKA) tetramer. Inactivating mutations of *PRKAR1A* lead to dissociation of the regulatory from the catalytic subunit, resulting in aberrant activity of PKA and phosphorylation of downstream targets, leading to cell proliferation and tumor formation [53].

Amongst the common endocrine abnormalities described in CNC are pituitary hyperplasia and PAs that are often associated with GH and/or PRL excess [56]. Although definite adenomas are present in 15% of patients, abnormal GH response to various stimuli, such as glucose or TRH, is present in almost 75% of patients [57,58]. Pituitary involvement in CNC is thought to be a progressive disorder with normal pituitary tissue progressing to somato(mammo)troph hyperplasia and subsequently, to distinct tumor formation [58]. Thus, multiple adenomas may be present synchronous or metachronous in the same patient [59]. Once diagnosed, GH excess is usually slowly progressive and although transsphenoidal resection of PAs may be attempted, there is high risk for recurrence. Medical therapy is often offered to the patients and sometimes, partial or complete hypophysectomy may be needed for control of disease [58,59].

Although CNC was thought to be associated almost exclusively with GH and /or PRL excess, most recently, two patients with Cushing disease (CD) have also been described, showing that other pituitary cell lineages may be rarely affected [60,61]. Somatic *PRKAR1A* gene changes are not reported in sporadic PAs [62,63].

### 2.4. McCune-Albright Syndrome (MAS)

The classic presentation of MAS (OMIM#174800) includes the triad of polyostotic fibrous dysplasia (FD), café-au-lait pigmentation and precocious puberty [64]. The disease is the result of postzygotic activating mutations of the *GNAS1* gene product, the cAMP-regulating protein Gsalpha (Gs_α_) [65]. It is now well recognized that additional endocrine overactivity disorders, other than precocious puberty, are part of the condition, including GH excess. GH excess in MAS presents in almost 21% of patients and may contribute to worsening of FD, vision and hearing loss, and macrocephaly [66]. For that reason, prompt medical therapy is recommended in all patients, while surgical intervention is not frequently attempted due to thick bones, the potential for worsening of skull FD, as well as the need for partial or total hypophysectomy [64].

### 2.5. X-Linked Acrogigantism (X-LAG)

X-LAG (OMIM#300942) has been recently described in patients with early onset GH excess [67]. X-LAG is caused by germline or somatic mosaic microduplications of Xq26.3, involving a G-protein-coupled receptor gene (*GPR101*) [68,69].

In a large study of 143 pediatric patients with gigantism, 10% of patients had microduplication of Xq26.3, two in familial cases and ten in sporadic adenomas [70]. Compared to patients without an identified genetic etiology, patients presented younger than the remaining patients, were more likely to co-secrete PRL, and had less risk of invasion of surrounding tissues [70]. In a cohort of 18 patients with X-LAG, mean age at diagnosis was 41 months and most patients had a macroadenoma identified on MRI, although no giant adenoma (by criteria of diameter more than 4 cm) was reported [67]. X-LAG is generally recognized as an aggressive disease given the difficulty to control GH excess. Most of the patients require multiple interventions (surgical and medical), and sometimes, subtotal or total hypophysectomy may be necessary. Radiation therapy is not generally helpful [67,71].

### 2.6. 3 P Association (3PAs)

The three P association or 3PAs is another recently identified condition involving the combination of pituitary adenomas, pheochromocytomas (PHEO) and/or paragangliomas (PGL) [72]. Xekouki et al. described that up to 75% of familial cases with 3PAs harbor pathogenic variants in the *SDHx* (*SDHB* and *SDHD*) genes [72]. Succinate dehydrogenase is part of the mitochondrial complex II and plays a significant role in energy production through the Krebs cycle and the respiratory chain through electron transfer [73]. Additional studies have further contributed in the genetic spectrum of the disease, by identifying additional genetic causes of the association (*SDHA*, *SDHAF2*, *VHL*, *MEN1*, *RET* and *MAX*) [74,75,76,77,78]. The presenting pituitary tumors are more commonly PRL- or GH-secreting or non-functioning adenomas, but they are thought to be more aggressive in presentation and more likely to be resistant to standard therapy [77].

### 2.7. DICER1

*DICER1* codes for a small RNA processing endoribonuclease that cleaves double stranded RNA into small interfering RNAs and mature miRNAs [79]. DICER1 or pleuropulmonary syndrome (OMIM#601200) is an autosomal dominant pleuropulmonary blastoma, familial tumor and dysplasia syndrome, which involves various tumors, such as pleuropulmonary blastomas, cystic nephromas, Sertoli-Leydig cell tumors, multinodular goiter and other [80].

A rare but almost pathognomonic manifestation of DICER1 syndrome is pituitary blastoma (PitB). PitBs differ from PAs in terms of their histological characteristics (since these are embryonic tumors) but given the potential for hormone production, they should be considered in the context of pituitary tumors. ACTH-secreting PitBs, although extremely rare, are almost always associated with *DICER1* mutations [81,82]. They are always diagnosed in infants and toddlers, less than 24 months of age [82]. Their management involves, in most cases, surgical removal, while at least one of the patients was treated with TMZ and radiation therapy. Four of the eleven reported patients died from complications of their disease, while at least two patients experienced recurrence [82]. Since PitBs are a rare association of DICER1 syndrome, routine screening with imaging or biochemical studies is not currently recommended for patients with DICER1 syndrome [83].

Recently, a case of isolated PRL-secreting adenoma in a 50-year old patient has been reported. Although histologic evaluation of the identified sellar tumor was not available, and the patient did not respond to medical therapy, this report expands the potential implications of DICER1 in adult-onset pituitary tumorigenesis [84].

### 2.8. Tuberous Sclerosis (TSC)

TSC is an autosomal dominant syndrome caused by genetic defects of *TSC1* (OMIM#191100) or *TSC2* (OMIM#613254) genes, which code for hamartin and tuberin respectively. TSC1/TSC2 mediate PI3K/Akt activation and lead to inhibition of the mTOR pathway [85].

TSC is characterized by hamartomas, epilepsy and mental retardation. Four patients with possible TSC and PAs have been described to date, ACTH-secreting (*n* = 2), GH-secreting (*n* = 1) and silent gonadotroph (n = 1) adenomas, suggesting that PAs may be a rare presentation of TSC [86,87,88,89].

### 2.9. Less Common Germline Genetic Defects Potentially Associated with PAs

NF1 (OMIM#613675) is an autosomal dominant syndrome caused by mutations or deletions in the *NF1* gene, reported in 1:3500 births [85]. *NF1* codes for neurofibromin which acts as a Ras-GTPase-activating protein; neurofibromin deficiency leads to constitutive activation of Ras-dependent pathways, namely Ras/Raf/MEK and Ras/PI3K/TSC/mTOR [85]. GH excess presents in 5.5–10.9% of patients with NF1 and optic pathway gliomas (OPGs), and it is thought to result from suppression of the hypothalamic somatostatin tone, while GHRH overactivity from OPGs has also been postulated in certain cases [90,91]. As a result, there is diffuse pituitary hyperplasia and no distinct adenoma is usually identified. Only one patient with a GH-secreting adenoma has been reported to date [92]. However, NF1 should remain in the differential diagnosis when evaluating a patient with acromegaly/gigantism.

Recently, Hernandez-Ramirez et al. described four potentially pathogenic variants in the *CABLES1* gene in patients with CD. CABLES1 interacts with cyclin-dependent kinase 3 and genetic defects lead to impaired inhibition of cell growth [93]. CNVs and methylation defects of other cyclin-dependent kinases (CDK2A and CDKN2C, coding for p16 and p18) have been reported in studies of PAs [94,95]. Additionally, a somatic mutation in *DKC1* gene, coding for dyskeratin, associated with X-Linked dyskeratosis congenita when present in germline, has been described in a patient with a non-functioning pituitary adenoma. The mutation is potentially associated with decreased p27 levels or defects in rRNA modification [96].

Finally, few patients with diagnosis of PA and additional genetic syndromes have been reported in the literature, for example, a patient with Beckwith-Wiedemann syndrome (loss of methylation of imprinting critical region 2 on chromosome 11p) and ACTH-secreting adenoma, two family members with Cantu syndrome (*ABCC9*) and non-functioning adenomas, a patient with autosomal dominant polycystic kidney disease (*PKD1*) and acromegaly, and a patient with X-linked adrenal hypoplasia (due to a *DAX1* defect) and ACTH-secreting adenoma [97,98,99,100]. The etiologic association of the pathogenesis of PA with the identified germline genetic defect is not confirmed in most of them, however, potential relationship cannot be excluded.

## 3. Somatic Changes

As mentioned above, somatic changes of genes involved in identified syndromes when present in germline may be identified exclusively in the tumor level in certain cases. For example, *GNAS* mutations are present in up to 50% of GH-secreting adenomas. Below we present genetic defects reported mainly in the tumor level of PAs.

### 3.1. Ubiquitin Specific Peptidase 8 (USP8)

USP8 is involved in the deubiquitination process of epidermal growth factor receptor (EGFR). Gain-of-function *USP8* gene defects lead to increased EGFR levels. In corticotroph cells, this defect results in high *POMC* expression and ACTH secretion [101,102]. Somatic defects of *USP8* represent the most frequent genetic defect in CD, present in approximately 20–60% of all ACTH-secreting adenomas [101,102,103,104,105,106]. However, they are uncommon in other PAs. Of note, all *USP8* somatic mutations associated with CD are located in the 14-3-3 binding motif (between amino acids 713 and 720) [101,102].

The prognosis of patients with somatic *USP8* mutations differs in various studies. Some reports of increased risk for recurrence of patients with somatic *USP8* mutations have not been replicated by others [104,106,107]. However, the potentially aggressive behavior of this genetic defect is evident in the patient with germline *USP8* defect, described recently by Cohen et al. [108]. The patient had severe recurrent CD difficult to control with surgical and medical therapies [108].

### 3.2. USP48 and BRAF

With the understanding of the importance of USP8 in corticotropinomas, further studies for other genes related to the MAPK pathway have been published. Recently, *USP48* and *BRAF* have been reported as additional target genes, with somatic variants of *BRAF* gene in 16% of patients and of *USP48* gene in 23% of *USP8*-negative corticotropinomas [109]. Of interest, variants in these genes were not detected in other pituitary tumors, suggesting a specific defect in the POMC regulation [109].

### 3.3. GNAS

Somatic *GNAS* mutations are common in sporadic GH-secreting adenomas presenting in up to 50% of these tumors [110,111]. Mutations are usually located at codons 201 and 227, and lead to activation of the G_sa_ subunit of *GNAS*, leading to increased adenylate cyclase activity, similarly to the defect seen in patients with MAS. Because of paternal imprinting of *GNAS* in the pituitary gland, mutations need to occur at the maternal allele to result in pituitary tumorigenesis [112]. A limited number of ACTH-secreting adenomas with somatic *GNAS* gene defects has been reported, showing that there may be a rare association with CD [113,114].

### 3.4. The Phosphoinositide 3-Kinase (PI3K)/AKT Pathway

The PI3K/AKT pathway regulates several cellular functions, including cell survival, growth, proliferation and metabolism [115]. Gene defects, including pathogenic variants and amplifications, in *PIK3CA* have been isolated in several cancers [116]. Studies in PAs identified a potential role in pituitary tumorigenesis as well with a frequency of somatic defects in 2.3–12.1% of all tumors, with higher incidence in invasive PAs [117,118].

### 3.5. The p53 Tumor Suppressor Gene

*TP53* codes for a tumor suppressor protein that has important implication in human carcinogenesis. Although p53 expression in pituitary tumors is associated with more aggressive behavior, several studies have failed to show a significant role of genetic defects of *TP53* in pituitary tumorigenesis [119,120,121]. Few cases of atypical PAs and pituitary carcinomas with somatic *TP53* defects have only been reported to date [122,123,124].

### 3.6. Copy Number Variations (CNVS) at the Chromosomal Level

Several studies have investigated the presence of CNVs in PAs. Although no recurrent CNV has been clearly noted, CNVs in chromosomes 1p and 11p have been reported in several occasions [125,126].

Recently, Bi et al. described a different profile of genomic aberrations: in a cohort of 42 PAs, a group of “disrupted” tumors, defined as large CNVs encompassing on average 39% of the tumor genome, were noted. These tumors were more likely to be functional adenomas, without evidence of more aggressive behavior [127,128]. Similar results were further described by other groups in GH-secreting tumors [129,130]. Our group also supported these studies by identifying a subgroup of patients with CD that harbored large genomic aberrations, involving up to 59% of the tumor genome. These tumors were associated with larger size and higher risk for cavernous sinus invasion [131].

## 4. Other Genomic and/or Molecular Events Associated with Various Features of PAs

### 4.1. Epigenetic Modifications of Pituitary Adenomas

The understanding of pathogenesis, differentiation and presentation of PAs has been further advanced through studies of epigenetic modifications.

Genome-wide methylation analyses of PAs have identified changes in methylation profile between normal pituitary tissue and PAs and attempted to clarify the differences between various subtypes of PAs [132,133]. Although the results have not been always consistent, changes in the methylation pattern of tumor suppressor and cell-cycle regulating genes (such as CASP8, tp73, RASSF1, Rb, p16, p18, SFN and other) or genes involved in the behavior of the tumors and their potential to invade tissues, like cell adhesion genes have been described [95,133,134,135,136]. Methylation changes also correlate with certain tumor characteristics, such as their size, functional status, and invasion of surrounding tissues, but again, results have not been replicated in all studies [94,133,135,137,138,139].

Growth factors have also been studied in relation to pituitary development and tumorigenesis [140]. FGFR2, and specifically, the predominat isoform FGFR2-IIIb) is downregulated in half of the pituitary adenomas, possibly due to increased promoter methylation, with potential implication for the expression of certain cyclin dependent kinases (such as p21 and p27) [141]. Furthermore, FGFR2 interacts with melanoma-associated antigen-3 (MAGE-A3) complex which further regulates p53 expression and leads to disordered cell cycle regulation [142]. FGFR4, on the other hand, is overexpressed in PAs, with potential implication in tumorigenesis [143].

Histone modifications and chromatin changes may also explain some of the PA characteristics, leading to altered expression of important cell-cycle genes (like Rb, p21 and p27) [142]. Examples of histone modifications and chromatin remodeling include the case of Ikaros, where expression of a certain isoform results in histone 3 acetylation and activation of the Bcl-XL promoter, leading to increased survival of pituitary cells [144,145].

### 4.2. miRNAs

miRNAs are small non-coding RNAs that are involved in cell cycle regulation and cancer pathogenesis via control of expression of various genes [146,147]. Over the last 10 years, studies on differentially expressed miRNAs in PAs compared to normal pituitary tissues have identified miRNAs with potential interest. For example, downregulation of miR-326, miR-432, miR-570, and miR-603, has been described in GH-secreting adenomas. These miRNAs potentially target the *HMGA2* and *E2F1* genes and their downregulation leads to increased expression and results in increased cell proliferation [148]. Several additional miRNAs, either upregulated or downregulated targeting genes affecting cell proliferation, apoptosis, invasion and even response to therapy, have been described, with potential implications for diagnostic, prognostic and therapeutic purposes [149,150,151].

## 5. Conclusions

The genomic landscape of PAs remains complex. Germline and somatic genetic defects contribute to our understanding of their pathogenesis, but do not explain the majority of them. Additional information derived from other gene regulation mechanisms should be considered in combination to describe the full picture of pituitary tumorigenesis.

## Figures and Tables

**Table 1:** Familial syndromes associated with pituitary adenomas (PAs). GH: growth hormone, PRL: prolactin, ACTH: Corticotropin hormone.

Familial Syndrome	Gene	Chromosomal Locus	Suggested Mechanism of Pituitary Tumorigenesis	Most Common Functional Status	Frequency of PAs
Familial Isolated Pituitary Adenomas	*AIP* (15–30% of cases)	11q13.2	Interaction in cAMP synthesis	GH	100%
Multiple Endocrine Neoplasia type 1	*MEN1*	11q13.1	Tumor suppressor; Involved in cell proliferation, genome stability and gene transcription	PRL or non-functioning	40%
Multiple Endocrine Neoplasia type 2A/2B	*RET*	10q11.21	Proto-oncogene; Transmembrane receptor with tyrosine kinase activity	Rare	Rare
Multiple Endocrine Neoplasia type 4	*CDKN1B*	12p13.1	Tumor suppressor; Cell cycle regulation	Rare	Rare
McCune-Albright	*GNAS*	20q13.32	cAMP-regulating protein Gsa; activation leads to increased cAMP levels and activation of protein kinase A (PKA)	GH excess	Up to 20%
Carney complex	*PRKAR1A*	17q24.2	Alpha regulatory subunit of PKA; inactivation of PRKAR1A leads to dissociation of the regulatory from the catalytic subunit and aberrant PKA activity	GH	15%
DICER1	*DICER1*	14q32.13	RNA processing endoribonuclease that cleaves double stranded RNA into small interfering RNAs and mature miRNAs	ACTH	Rare
3 P association	*SDHx*, *VHL*, *MEN1*, *RET* and *MAX*	5p15.33 (*SDHA*) 1p36.13 (*SDHB*) 11q23.1 (*SDHD*) 11q12.2 (*SDHAF2*) 3p25.3 (*VHL*) 11q13.1 (*MEN1*) 11q13.1 (*RET*) 14q23.3 (*MAX*)	Several functions depending on identified gene. SDHx: participate in the mitochondrial complex II, energy production through the Krebs cycle and respiratory chain through electron transfer. VHL: tumor suppressor; oxygen sensing and interaction with hypoxia-inducible factors. MAX: Myc associated factor X; involved in cell proliferation, differentiation and apoptosis	PRL or GH	100%
Tuberous sclerosis	*TSC1*, *TSC2*	9q34.13 (*TSC1*) 16p13.3 (*TSC2*)	Mediate PI3K/Akt activation and lead to inhibition of mTOR pathway	ACTH	Rare
X-Linked Acrogigantism	*GPR101*	Xq26.3	G-protein-coupled receptor; defects lead to constitutive activation of the cAMP-PKA pathway	GH	85%
Neurofibromatosis type 1	*NF1*	17q11.2	Ras-GTPase-activating protein involved in Ras-dependent pathways (Ras/Raf/MEK and Ras/PI3K/TSC/mTOR)	GH	Rare

**Table 2:** Clinical presentation of patients with familial syndromes involving PAs. MEN: Multiple Endocrine Neoplasia, GH: growth hormone, ACTH: Corticotropin hormone.

Familial Syndrome	Gene	Presentation
Familial Isolated Pituitary Adenomas	*AIP* (15–30% of cases)	Presence of at least two family members with pituitary adenomas, either of the same functional status (homologous) or of different (heterologous), without extra-pituitary findings.
Multiple Endocrine Neoplasia type 1	*MEN1*	Autosomal dominant syndrome presenting with multiple endocrine neoplasias, including anterior pituitary adenomas, hyperparathyroidism, enteropancreatic tumors, neuroendocrine tumors and others.
Multiple Endocrine Neoplasia type 2A/2B	*RET*	Autosomal dominant syndromes presenting with medullary thyroid carcinoma (MEN2A/B), pheochromocytoma (MEN2A/B), hyperparathyroidism (MEN2A), mucosal ganglioneuromas (MEN2B), and rare occurrence of anterior pituitary adenomas.
Multiple Endocrine Neoplasia type 4	*CDKN1B*	Autosomal dominant MEN1-like syndrome, without genetic confirmation of *MEN1* gene defect.
McCune-Albright	*GNAS*	Classic triad of polyostotic fibrous dysplasia, café-au-lait macules, and precocious puberty. Additional features include GH excess, hyperthyroidism, neonatal Cushing syndrome, and hypophosphatemia.
Carney complex	*PRKAR1A*	Autosomal dominant syndrome presenting with the constellation of cardiac and skin myxomas, spotty skin pigmentation, endocrine overactivity, including anterior pituitary adenomas and ACTH-independent Cushing syndrome, breast and testicular tumors, thyroid nodules, psammomatous melanotic schwannomas, and osteochondromyxomas.
DICER1	*DICER1*	Autosomal dominant syndrome presenting with pleuropulmonary blastomas, cystic nephromas, Sertoli-Leydig cell tumors, multinodular goiter, pituitary blastomas and other tumors.
3 P association	*SDHx*, *VHL*, *MEN1*, *RET* and *MAX*	Combination of pituitary adenomas, pheochromocytomas and/or paragangliomas.
Tuberous sclerosis	*TSC1*, *TSC2*	Autosomal dominant syndrome characterized by hamartomas, epilepsy, mental retardation, and rare occurrence of pituitary neuroendocrine tumors
X-Linked Acrogigantism	*GPR101*	GH excess with onset of symptoms in most cases younger than 2 years of age.
Neurofibromatosis type 1	*NF1*	Autosomal dominant syndrome presenting with neurofibromas, skin findings (café-au-lait macules and freckling), Lisch (iris) nodules, optic pathway gliomas with consequent precocious puberty and GH excess, and other rare tumors.

**Table 3:** Commonest somatic genetic defects associated with PAs. GH: growth hormone, PKA: protein kinase A, EGFR: Epidermal growth factor receptor, ACTH: Corticotropin hormone.

Gene	Chromosomal Locus	Suggested Mechanism of Pituitary Tumorigenesis	Most Common Functional Status
*GNAS*	20q13.32	cAMP-regulating protein Gsa; activation leads to increased cAMP levels and activation of protein kinase A (PKA)	GH
*USP8*	15q21.2	Involved in deubiquitination of EGFR; gain of functions mutations cause increase EGFR, and POMC expression	ACTH
*AIP*	11q13.2	Interaction in cAMP synthesis	GH
*USP48*	1p36.12	Deubiquitination; activation of MAPK and increased POMC expression	ACTH
*BRAF*	7q34	Proto-oncogene with tyrosine kinase activity; activation of MAPK and increased POMC expression	ACTH
*PIK3CA*	3q26.32	Involved in PI3K/AKT pathway which regulates several cellular functions, including cell survival, growth, proliferation and metabolism	Non-functioning
*TP53*	17p13.1	Tumor suppressor; involved in cell cycle, apoptosis and genomic stability	ACTH(also associated with atypical adenomas and pituitary carcinomas)
